# Synergistic Microbial Consortium for Bioenergy Generation from Complex Natural Energy Sources

**DOI:** 10.1155/2014/139653

**Published:** 2014-07-06

**Authors:** Victor Bochuan Wang, Joey Kuok Hoong Yam, Song-Lin Chua, Qichun Zhang, Bin Cao, Joachim Loo Say Chye, Liang Yang

**Affiliations:** ^1^Singapore Centre on Environmental Life Sciences Engineering (SCELSE), Nanyang Technological University, Singapore 637551; ^2^School of Materials Science and Engineering, Nanyang Technological University, Singapore 639798; ^3^Interdisciplinary Graduate School, Nanyang Technological University, Singapore 637551; ^4^Graduate School of Integrative Sciences and Engineering, National University of Singapore, Singapore 117543; ^5^School of Civil and Environmental Engineering, Nanyang Technological University, Singapore 639798; ^6^School of Biological Sciences, Nanyang Technological University, Singapore 637551

## Abstract

Microbial species have evolved diverse mechanisms for utilization of complex carbon sources. Proper combination of targeted species can affect bioenergy production from natural waste products. Here, we established a stable microbial consortium with *Escherichia coli* and *Shewanella oneidensis* in microbial fuel cells (MFCs) to produce bioenergy from an abundant natural energy source, in the form of the sarcocarp harvested from coconuts. This component is mostly discarded as waste. However, through its usage as a feedstock for MFCs to produce useful energy in this study, the sarcocarp can be utilized meaningfully. The monospecies *S. oneidensis* system was able to generate bioenergy in a short experimental time frame while the monospecies *E. coli* system generated significantly less bioenergy. A combination of *E. coli* and *S. oneidensis* in the ratio of 1 : 9 (v : v) significantly enhanced the experimental time frame and magnitude of bioenergy generation. The synergistic effect is suggested to arise from *E. coli* and *S. oneidensis* utilizing different nutrients as electron donors and effect of flavins secreted by *S. oneidensis*. Confocal images confirmed the presence of biofilms and point towards their importance in generating bioenergy in MFCs.

## 1. Introduction

Unprecedented industrialization and the continued spurt in population growth have vastly depleted global natural energy sources. This has led to an acute need for alternative, clean, and renewable energy sources. In particular, extensive efforts have been invested into increasing efficiencies of solar cells [1], which has been envisioned as the next frontier in renewable energy. Another potential source of alternate energy lies in producing bioenergy from agricultural waste products via microbial activity.

One potential approach to producing bioenergy from natural waste products is through microbial fuel cells (MFCs) which employ the extracellular electron transport (EET) functionalities of electrochemically active bacteria (EAB) to facilitate electron transport and thus produce electricity from diverse energy sources [2]. The free electrons and protons originate from microbial metabolism of organic components found in the media within an anaerobic anode chamber. Metabolism is achieved when electrons move along the cascading energy pathway of the electron transport chain, which releases energy for continued survival of the microorganism. These electrons are further transported by various EET mechanisms to the external terminal electron acceptors. A voltage is generated in the process of electrons moving across the external resistor towards the cathode. Protons diffuse simultaneously across the selective proton exchange membrane to the aerobic cathode chamber. In this compartment, oxygen is reduced by electrons and protons to produce water molecules in order to complete the charge balance. Although this technology has matured over time and shows promise in concurrent bioremediation and power generation [3], it has seen little commercial success. This is due to high material cost and low power performance that is partly caused by limitations in inferior charge transport at the inherently insulating microbe-electrode interface. Much effort has been invested in circumventing these bottlenecks. Recently, enhanced power output in MFCs has been demonstrated through chemical modification of the insulating interface junction across* Escherichia coli* cellular membrane [4, 5] and genetic engineering of* Pseudomonas aeruginosa* to enhance endogenous secretion of pyocyanin mediators [6]. Further, small-scale stacked MFCs have been shown to power mobile devices using human urine as an energy source [7]. Better understanding of microbial species interactions employing EET processes has been proposed as a promising strategy to improve the performance of MFCs [8, 9].

In this contribution, a synergistic microbial consortium was established and modified for bioenergy generation from a complex energy source, in the form of the coconut sarcocarp, which is defined as the fleshy part of the fruit. According to statistics from the Food and Agriculture Organization of the United Nations, ~54 million tonnes of coconut were produced in 2010 from mainly tropical coastal countries, of which a large amount is wasted [10, 11]. However, coconuts are known to be rich sources of sugars, fats, oil, and carbohydrates with small beneficial concentrations of vitamins and salts [12]. Hence, coconuts which are considered as waste products can be potentially used as an alternative and natural energy source for tropical coastal countries.

By choosing the model non-EAB (*E. coli*) and the EAB (*Shewanella oneidensis*), we demonstrate that modification of the ratio of bacterial strains introduced into the microbial consortium can significantly improve MFC performance.

## 2. Materials and Methods

### 2.1. Media Preparation and Bacterial Strains

The sarcocarp from a fresh coconut was removed and homogenized using a Bio-Gen PRO200 Homogenizer (PRO Scientific Inc, USA) at maximum speed for 5 min. All parts of the equipment were dismantled and wiped down with 70% ethanol to adhere to sterility requirements. Further, the homogenization process took place inside a sterile biosafety cabinet to avoid contamination. The resulting slurry was further diluted 2x with sterile deionized water to form the medium for dispensing into the MFCs. Monocultures of* E. coli* (red fluorescent protein (RFP) tagged) and* S. oneidensis* (green fluorescent protein (GFP) tagged) [13] were grown aerobically overnight in lysogeny broth (LB) at 37°C and 30°C, respectively, while shaking at 200 rpm.

### 2.2. Setup of MFCs

All materials were used as received, unless otherwise stated. Dual-chamber MFCs were constructed as previously described [3, 4, 6]. 19 mL of diluted sarcocarp slurry was dispensed into the anode chamber, prior to inoculation of the bacterial strains. 1 mL of culture (OD_600_~1.0) for each bacterial strain was then inoculated into the anode chamber only. Final volume of each chamber is maintained at 20 mL. The incubator housing of the MFCs was set to 33°C. Data recording started immediately after inoculation. Glass tubes (17 mm O.D. × 1.8 mm wall thickness) forming the anode and cathode chambers of the MFCs, carbon felt (3.18 mm thickness), and stainless steel pinch clamps (#28) were purchased from VWR Pte. Ltd. Titanium wire (0.25 mm diameter), Nafion N117 proton exchange membrane (PEM), and serrated silicone septa (18 mm O.D.) were purchased from Sigma-Aldrich. Nylon screws and nuts were purchased from Small Parts, Inc. 90° O-ring-groove-to-plain-end glass tubes were separated from each other by a piece of Nafion N117 proton exchange membrane. The joints of the glass tubes were greased and sealed against a circular piece of Nafion membrane (diameter of 2 cm). The whole assembly was held in place and tightened with a stainless steel pinch clamp. Carbon felt electrodes were cut to 2 cm × 5 cm dimensions (width × length) and connected to the titanium wire via the screws and nuts. The electrodes were then seated inside the glass tubes. Prior to MFC operation, the devices were filled with ultrapure water and autoclaved to sterilize the internal components in the devices. After sterilization, the water was dispensed and diluted sarcocarp slurry was introduced to both chambers. The anode chamber was sealed with a silicone septum through which the titanium wire was threaded, while the cathode chamber was loosely capped with an inverted glass scintillation vial to provide an aerobic environment. The cathode electrodes were only partly submerged in the catholyte to allow for an “air-wicking” aerobic configuration. The electrodes were then connected to a 1 kΩ resistor and voltage measurements across the resistors were recorded at a rate of 1 point per 5 minutes using an eDAQ e-corder data acquisition system (Bronjo Medi) equipped with Chart software. Voltage readings are collected as raw data and further converted to current density for presentation. Current is calculated according to the following equation:

(1) $$\begin{array}{c} I=\frac{V}{R}, \end{array}$$

where *I* is the current in amperes (A), *V* is the potential difference in volts (V), and *R* is the resistance in ohms (Ω). Current density is obtained by dividing the equation above by the geometrical surface area of the electrode (by 20 cm^2^).

### 2.3. Biofilm Imaging

Electrodes from the anode chamber were removed from the corresponding MFCs. All microscopy images of RFP-tagged* E. coli* and GFP-tagged* S. oneidensis* biofilms formed on the electrode surface were acquired by Carl Zeiss Confocal Laser Scanning Microscope (CLSM model LSM 780) (Carl Zeiss, Germany) with 40x objective lens after mounting the electrode fibers onto microscope slides. Image processing was performed with the software package, Zen 2011, provided by Carl Zeiss.

## 3. Results and Discussion

### 3.1. Electrical Performance

**Figure 1:**
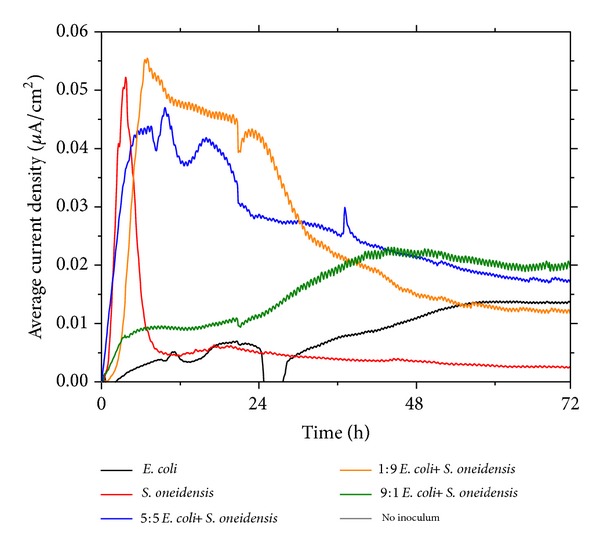
Average current density versus time of MFCs with various bacterial species and ratios.

Dual-chamber MFCs were employed to investigate the bioenergy generated as the coconut sarcocarp is broken down through microbial oxidation by the respective bacterial species. The average current densities generated over 72 h were recorded (Figure 1). The consistently low current densities from all operated MFCs were attributed to high internal resistances within the bioelectrochemical devices, which impede charge movement. Further, the media in the anode and cathode chambers contained the sarcocarp slurry, which has limited conductivity. This can be averted through various forms of optimization, such as adopting different device architectures [14], apparatus components, or electrode engineering [15]. However, the focus of this study was to demonstrate facile bioenergy generation through the use of a natural, abundant, and readily available energy source, coconut sarcocarp, by employing common bacterial species. MFCs inoculated with* E. coli* generated an average maximum current density of ~0.015 *μ*A/cm^2^ (Figure 1, black trace), whereas* S. oneidensis* MFCs generated ~0.05 *μ*A/cm^2^ (Figure 1, red trace). The rapid decrease in average current density generated by the* S. oneidensis* MFCs after ~6 h is caused by the depletion of suitable energy sources available for* S. oneidensis*. This is because the single fed batch MFC system was employed in this study, which is in contrast to a continuous fed system, where the energy source can be renewed through a steady exchange of spent and fresh medium. The average current density generated by the* S. oneidensis* MFCs stabilized at a significantly lower current density of ~0.005 *μ*A/cm^2^ up to 72 h. MFCs without any inoculum were also operated and negligible current density was generated (Figure 1, grey trace). This indicates that the observed current densities were driven by the microbial actions of* E. coli* and* S. oneidensis* mono- and cocultures on the sarcocarp.

It has been well established that electrochemically active* S. oneidensis* has various forms of EET mechanisms, such as conducting outer membrane cytochromes [16], nanoappendages [17], and secretion of flavins [18], which act as charge transport mediators. These mechanisms are electrical conduits to transfer microbially released electrons to terminal electron acceptors. The ~3-fold difference in average maximum current density from monoculture systems is attributed to poorly evolved* E. coli* EET mechanisms which lack the diversity and effectiveness of EET mechanisms in* S. oneidensis*. Notably, significant bioenergy generation by* S. oneidensis* started from an early stage, while output from* E. coli* only started to increase later. This suggests that* S. oneidensis* and* E. coli* might utilize different energy sources present in the sarcocarp for bioenergy generation. It is further hypothesized that, in coculture MFCs containing* E. coli* and* S. oneidensis*, a possible synergistic effect involving flavins has been created. To test this hypothesis, coculture systems utilizing various ratios of* E. coli* and* S. oneidensis* were operated to investigate possible synergistic interactions. Interestingly, a 5 : 5 (50% : 50%, v : v) coculture system produced a maximum current density of ~0.045 *μ*A/cm^2^ (Figure 1, blue trace). As compared to the monoculture systems (Figure 1, red trace for* S. oneidensis*, black trace for* E. coli*), the 5 : 5 coculture system could generate a relatively sustainable and significant current density over 72 h. It is thus noteworthy to further probe the effect of different bacterial ratios on the extent of bioenergy generation. A 1 : 9 (v : v)* E. coli* and* S. oneidensis* system generated a maximum current density of ~0.055 *μ*A/cm^2^ (Figure 1, orange trace), whereas a 9 : 1 (v : v)* E. coli* and* S. oneidensis* system generated a maximum current density of ~0.025 *μ*A/cm^2^ (Figure 1, green trace). The ratio modification study suggests that introducing a higher concentration of* S. oneidensis* in the coculture systems allows for maximum exploitation of suitable energy sources for* S. oneidensis*. This strategy may minimize consumption of such energy sources by* E. coli*, which generates significantly lesser bioenergy, and facilitate generation of excess secreted flavins to enhance* E. coli* bioenergy generation at a later stage when the species uses suitable energy sources for itself.

### 3.2. Biofilm Characterization

**Figure 2:**
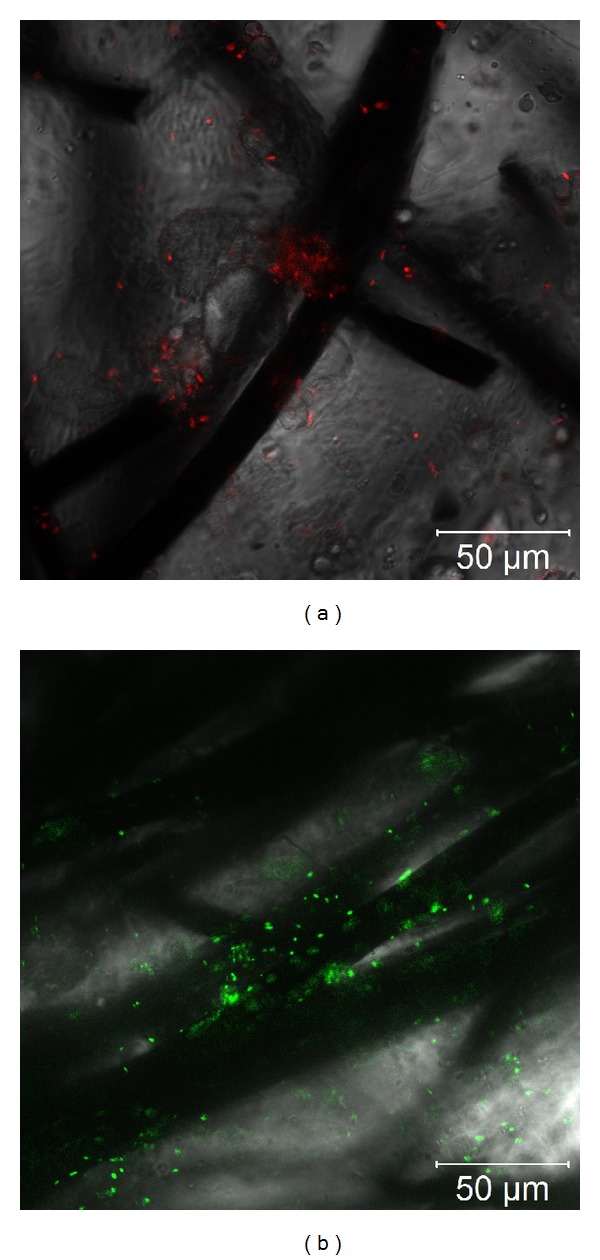
Overlaid brightfield and confocal microscopy images of stained biofilms on respective electrodes. (a)* E. coli* biofilm. (b)* S. oneidensis* biofilm.

Further, the role of biofilms in the bioelectrochemical systems was elucidated by confocal microscopy characterization. Representative overlaid brightfield and confocal images were acquired from random strands of electrodes in respective MFCs. Biofilms were formed in all systems (Figures 2(a) and 2(b)). To differentiate between each species,* E. coli* was tagged with red fluorescent protein (RFP), whereas* S. oneidensis* was tagged with green fluorescent protein (GFP). The RFP-tagged* E. coli* biofilm and GFP-tagged* S. oneidensis* biofilm were evident on the electrode fiber surfaces (Figures 2(a) and 2(b)). The confocal images corroborate the importance of the biofilm in the electrical performances with specific bacterial strains.

### 3.3. Mechanistic Insights of the Functional Coculture System

**Figure 3:**
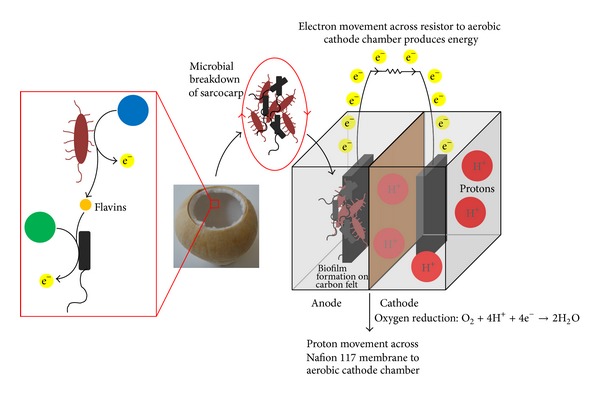
Diagram illustrating mechanistic reactions in coculture MFCs. Black schematic depicting nonelectrochemically active microorganisms, such as* E. coli*; red schematic depicting electrochemically active microorganisms, such as* S. oneidensis*; blue and green schematics depicting energy sources most favourable for breakdown by electrochemically active and nonelectrochemically active microorganisms, respectively.

The following possible mechanisms occurring in the coculture system were proposed (Figure 3). Various favourable nutrients (represented by blue and green dots) present in the sarcocarp can be broken down specifically by the independent microbial oxidative actions of non-EAB (*E. coli*) and EAB (*S. oneidensis*) in different stages of MFC operation to produce bioenergy. From the electrical data (Figure 2), it is suggested that, for significant and sustained bioenergy production, the EAB should be introduced at a higher concentration. This is to restrict nutrient consumption by non-EAB. The EAB also breaks down its suitable energy source and secretes flavins, which can be utilized by non-EAB at a later stage to facilitate EET. Gradual decline in current densities is due to lack of available nutrients in the closed system (Figure 1).

## 4. Conclusions

In summary, we have demonstrated bioenergy generation in MFCs by employing a natural and abundant feedstock, coconut sarcocarp. The common EAB,* S. oneidensis*, and the non-EAB,* E. coli*, were employed and a possible synergy was suggested, based on the ratio of microbial species introduced to the system. This demonstration paves the way forward for exploration of alternative and natural energy sources using mixed species consortia.

## References

[1] Xing G, Mathews N, Sun S (2013). Long-range balanced electron- and hole-transport lengths in organic-inorganic CH_3_NH_3_PbI_3_. *Science*.

[2] Lovley DR (2008). The microbe electric: conversion of organic matter to electricity. *Current Opinion in Biotechnology*.

[3] Wang VB, Chua S-L, Cai Z (2014). A stable synergistic microbial consortium for simultaneous azo dye removal and bioelectricity generation. *Bioresource Technology*.

[4] Wang VB, Du J, Chen X (2013). Improving charge collection in *Escherichia coli*-carbon electrode devices with conjugated oligoelectrolytes. *Physical Chemistry Chemical Physics*.

[5] Hou H, Chen X, Thomas AW (2013). Conjugated oligoelectrolytes increase power generation in *E. coli* microbial fuel cells. *Advanced Materials*.

[6] Wang VB, Chua S, Cao B (2013). Engineering PQS biosynthesis pathway for enhancement of bioelectricity production in *Pseudomonas aeruginosa* microbial fuel cells. *PLoS ONE*.

[7] Ieropoulos IA, Ledezma P, Stinchcombe A, Papaharalabos G, Melhuish C, Greenman J (2013). Waste to real energy: the first MFC powered mobile phone. *Physical Chemistry Chemical Physics*.

[8] Du Z, Li H, Gu T (2007). A state of the art review on microbial fuel cells: A promising technology for wastewater treatment and bioenergy. *Biotechnology Advances*.

[9] Lovley DR (2006). Microbial fuel cells: novel microbial physiologies and engineering approaches. *Current Opinion in Biotechnology*.

[10] Sumathi S, Chai SP, Mohamed AR (2008). Utilization of oil palm as a source of renewable energy in Malaysia. *Renewable & Sustainable Energy Reviews*.

[11] Goh CS, Tan KT, Lee KT, Bhatia S (2010). Bio-ethanol from lignocellulose: status, perspectives and challenges in Malaysia. *Bioresource Technology*.

[12] Mourao DM, Bressan J, Campbell WW, Mattes RD (2007). Effects of food form on appetite and energy intake in lean and obese young adults. *International Journal of Obesity*.

[13] Zhang Y, Ng CK, Cohen Y, Cao B (2014). Cell growth and protein expression of *Shewanella oneidensis* in biofilms and hydrogel-entrapped cultures. *Molecular BioSystems*.

[14] Ringeisen BR, Henderson E, Wu PK (2006). High power density from a miniature microbial fuel cell using *Shewanella oneidensis* DSP10. *Environmental Science & Technology*.

[15] Zhang T, Zeng Y, Chen S, Ai X, Yang H (2007). Improved performances of *E. coli*-catalyzed microbial fuel cells with composite graphite/PTFE anodes. *Electrochemistry Communications*.

[16] Coursolle D, Baron DB, Bond DR, Gralnick JA (2010). The Mtr respiratory pathway is essential for reducing flavins and electrodes in *Shewanella oneidensis*. *Journal of Bacteriology*.

[17] El-Naggar MY, Wanger G, Leung KM (2010). Electrical transport along bacterial nanowires from *Shewanella oneidensis* MR-1. *Proceedings of the National Academy of Sciences of the United States of America*.

[18] Von Canstein H, Ogawa J, Shimizu S, Lloyd JR (2008). Secretion of flavins by *Shewanella* species and their role in extracellular electron transfer. *Applied and Environmental Microbiology*.

